# Epidemiology of canine gastrointestinal helminths in sub-Saharan Africa

**DOI:** 10.1186/s13071-018-2688-9

**Published:** 2018-02-20

**Authors:** Nozyechi Ngulube Chidumayo

**Affiliations:** Clinical Studies Department, School of Veterinary Medicine, University of Zambia, P. O. Box 32379 Lusaka, Zambia

**Keywords:** Dogs, Epidemiology, Helminths, Meta-analysis, Prevalence, Sub-Saharan Africa

## Abstract

**Background:**

Dogs have a close association with humans providing companionship, security and a source of dietary protein. However, dogs are also potential carriers of zoonotic pathogens. Dogs, therefore, pose a public health risk and a good understanding of canine diseases is important for planning and implementing control measures. The aim of this study was to characterise canine helminthiasis in sub-Saharan Africa using a systematic approach.

**Methods:**

Pubmed and Google Scholar were searched for relevant primary studies published from 2000. Forty-one eligible studies were included in the meta-analysis. Pooled prevalences were estimated using the quality effects model.

**Results and conclusions:**

Twenty-six genera of enteric helminths were reported and the pooled estimate of canine helminthiasis was 71% (95% CI: 63–79%). Species of *Ancylostoma* and *Toxocara*, causative agents of *larva migrans* in humans, were the most frequently reported helminths with pooled estimated prevalences of 41% (95% CI: 32–50%) and 22% (95% CI: 16–29%), respectively. *Dipylidium caninum* and *Taenia* spp. were the most frequently reported cestodes with pooled estimated prevalences of 20% (95% CI: 12–29%) and 9% (95% CI: 5–15%), respectively. Trematodes were rarely reported. There was a high level of heterogeneity in most pooled estimates (I^2^ ˃ 80%). The results of this study show that canine helminthiasis is highly prevalent in sub-Saharan Africa and there is need for regular deworming programmes to improve the health status of the dogs and minimise the potential health risk to humans.

**Electronic supplementary material:**

The online version of this article (10.1186/s13071-018-2688-9) contains supplementary material, which is available to authorized users.

## Background

Dogs have a close association with humans providing companionship, security and protein [1]. However, dogs are also carriers of zoonotic pathogens including *Toxocara canis* and *Ancylostoma* spp. [1, 2]. *Toxocara canis* can cause diarrhoea, poor growth and death if present in large numbers in puppies [2]*. Ancylostoma* spp. are one of the most pathogenic helminths in dogs, especially puppies [2]. These nematodes are hematophagous and can cause anaemia and death if present in large numbers [2].

Canine zoonotic helminths pose a public health risk through environmental faecal contamination [3–5]. Canine and human infection with zoonotic canine helminths can occur through ingestion of the infective eggs and ingestion or skin penetration of the infective larvae [1, 2]. Human infection with *Toxocara* spp. is typically asymptomatic, however, some individuals develop visceral *larva migrans* and ocular toxocariasis [1, 2, 6]. *Ancylostoma* spp. are the aetiological agents of cutaneous *larva migrans* [1, 2] and *Ancylostoma caninum* has also been associated with eosinophilic enteritis in humans [7–10]. It is therefore important to understand the epidemiology of helminth infections in dogs to improve animal health and prevent the spread of zoonotic pathogens to humans.

The aim of this study was to characterise canine helminthiasis in sub-Saharan Africa and identify the most commonly reported helminths using a systematic approach.

## Methods

### Literature search

Pubmed and Google Scholar were systematically searched for publications from 2000 using the following search terms: dogs helminth, dogs helminthes, dogs helminths, dogs endoparasites, dogs gastrointestinal parasites, dogs gastro-intestinal parasites, dogs intestinal parasites, dogs enteric parasites, dog helminth, dog helminths, dog helminthes, dog endoparasites, dog gastro-intestinal parasites, dog gastrointestinal parasites, dog intestinal parasites, dog enteric parasites, canine helminth, canine helminths, canine helminthes, canine endoparasites, canine gastrointestinal parasites, canine gastro-intestinal parasites, canine intestinal parasites canine, enteric parasites, dogs nematodes, dog nematodes, canine nematodes, dogs cestodes, dog cestodes, canine cestodes, dogs trematodes, dog trematodes and canine trematodes. Title/abstract searches in Pubmed identified 786 articles. Title searches in Google Scholar identified 261 articles. The last search was conducted on 10th September 2016. Articles were stored in EndNote X5 (Thomson Reuters, New York, USA).

### Eligibility criteria

The titles and abstracts were reviewed and articles were selected based on the following criteria: English language; full-text journal articles published from 2000; conducted in a country in sub-Saharan Africa; cross-sectional or prospective studies. Articles were excluded if they did not report prevalence data, were case-control studies, clinical trials or pharmacological studies.

### Quality of the studies

Eligible studies were assessed for quality of reporting and selection for bias using a quality assessment checklist [11, 12] (Additional file 1).

### Data extraction

The following data were extracted where possible: location of the study, sample size, overall prevalence, genus and prevalence of detected helminths, sampling method, type of sample collected, sample processing and diagnostic method, age and sex of the dogs. For simplicity, dogs were characterised as puppies (≤ 6 months), juveniles (˃ 6–12 months), immature (≤ 12 months) and mature (> 12 months). Relevant data was stored in Microsoft Excel.

### Data analysis

MetaXL version 3.1 (http://www.epigear.com/), a tool for meta-analysis in Microsoft Excel, was used to pool prevalences from each study [13, 14]. Pooled estimated prevalences and their 95% confidence intervals (CI) were calculated using the quality effects model. The quality effects model uses quality scores to weigh studies according to sample size and study quality [13]. Pooled prevalence estimates were calculated for genera where prevalence data was extracted from a minimum of five studies. Heterogeneity among studies was evaluated by I^2^. I^2^ values of 25%, 50% and 75% were considered as having a low, moderate and high degree of heterogeneity, respectively [15]. Publication bias was assessed using funnel plots and doi plots. The symmetry of the doi plots was evaluated using the LFK index. LFK index values within ± 1, exceeding ± 1 but within ± 2, and exceeding ± 2 were considered as having no asymmetry, minor asymmetry and major asymmetry, respectively [14].

Potential sources of heterogeneity were further assessed by arranging the studies in subgroups according to sex, age (puppy and juvenile or immature and mature) and geographical regions (East Africa, West Africa and southern Africa). Regional subgroup analysis was conducted for helminth genera reported in at least 50% of the articles.

## Results

### Characteristics of eligible studies

**Fig. 1:**
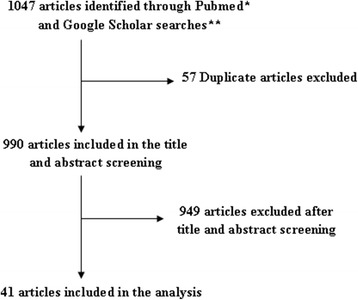
Flow chart of literature search and selection. *Title/abstract searches were conducted in Pubmed for articles published from 2000 to 2016. **Title searches were conducted in Google Scholar for articles published from 2000 to 2016. **The searches were limited to English pages and excluded patents and citations

A total of 41 articles [16–56] from eight countries were identified (Fig. 1) and included in this study. The sample size of individual studies ranged from 33 to 1000 dogs. The quality of the selected studies ranged from 1 to 9. Additional file 2: Table S1 and Additional file 3: Figure S1 give an overview of characteristics of eligible studies. A total of 12,029 samples, consisting of 11,717 faecal samples and 312 intestinal samples were included in the study. All samples were analysed using microscopy and processed using flotation (39%), sedimentation and flotation (39%), Kato-Katz (10%), sedimentation (5%) and washing and decantation (2%).

### Pooling and heterogeneity analyses

**Fig. 2:**
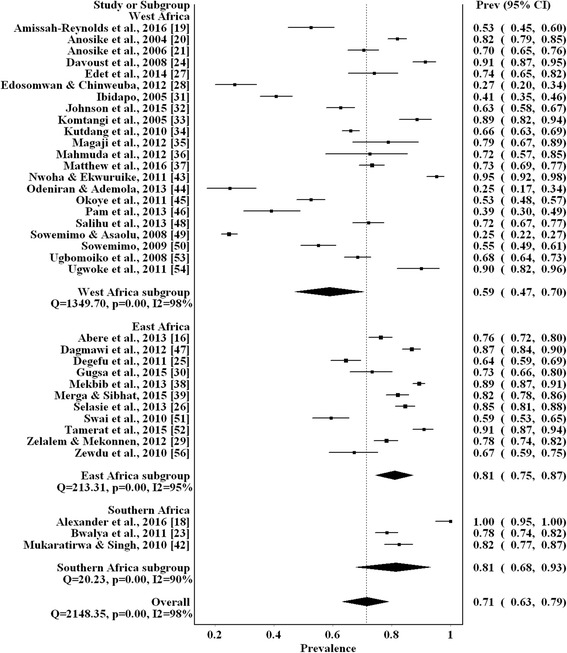
Forest plot of the prevalence estimates of canine gastrointestinal helminths

Thirty-six and 39 articles reported the overall and genera prevalence respectively. Twenty-six genera of helminths were reported across the studies (Additional file 4: Figure S2). Nematodes were the most frequently reported helminths followed by cestodes and trematodes. The overall prevalence of gastrointestinal helminths was 71% (95% CI: 63–79%) among 11,343 dogs (Table 1 and Fig. 2). Southern Africa and East Africa had the highest prevalence of 81% (95% CI: 68–93%) and 81% (95% CI: 75–87%), respectively, while West Africa had the lowest (59%; 95% CI: 47–70%). *Ancylostoma* spp. were the most prevalent helminths (41%; 95% CI: 32–50%), followed by *Toxocara* spp. (22%; 95% CI: 16–29%) and *Dipylidium caninum* (20%; 95% CI: 12–29%). Additional file 5: Figure S3 shows the forest plots of the estimated prevalences of the common helminth genera.

**Table 1:** Estimated pooled prevalences of canine gastrointestinal helminths in sub-Saharan Africa

Helminth	Region	Sample size	No. positive	Pooled prevalence	95% CI	I^2^ (%)
Overall prevalence	Sub-Saharan Africa	11,343	7791	71	63–79	98
	West Africa	6550	3934	59	47–70	98
	East Africa	4068	3272	81	75–87	95
	Southern Africa	725	585	81	68–93	90
*Ancylostoma* spp.	Sub-Saharan Africa	11,214	4669	41	32–50	99
	West Africa	5876	1882	28	18–39	98
	East Africa	4514	2251	49	36–61	98
	Southern Africa	824	536	66	37–93	97
*Toxocara* spp.	Sub-Saharan Africa	11,420	2720	22	16–29	98
	West Africa	6346	1563	21	11–32	99
	East Africa	4250	1050	24	16–33	97
	Southern Africa	824	107	12	3–24	90
*Dipylidium caninum*	Sub-Saharan Africa	9717	1904	20	12–29	99
	West Africa	5570	684	10	3–20	99
	East Africa	3563	1137	32	25–38	93
	Southern Africa	584	83	11	0–55	98
*Taenia* spp.	Sub-Saharan Africa	6224	661	9	5–15	97
	West Africa	3481	324	7	3–11	93
	East Africa	1919	309	14	2–30	99
	Southern Africa	824	28	2	0–11	96
*Strongyloides* spp.	Sub-Saharan Africa	5398	1345	23	11–38	99
*Uncinaria stenocephala*	Sub-Saharan Africa	2083	283	7	0–39	99
*Trichuris vulpis*	Sub-Saharan Africa	8692	883	5	2–10	98
*Spirocerca lupi*	Sub-Saharan Africa	2602	244	7	2–14	96
*Echinococcus* spp.	Sub-Saharan Africa	3087	162	5	3–7	84
*Toxascara* spp.	Sub-Saharan Africa	4040	192	3	1–7	95

*Abbreviation*: *CI* confidence interval

Sex and age prevalences are summarised in Table 2. The overall estimated pooled prevalence of gastrointestinal helminths in male dogs was 62% (95%: 45–78%) while the prevalence in female dogs was 56% (95% CI: 40–71%). Prevalence of helminths in immature and mature dogs were 74% (95% CI: 56–89%) and 62% (95% CI: 42–81%), respectively. Juveniles had lower overall estimated pooled prevalence compared to puppies. In addition, estimated pooled prevalences of *Ancylostoma* spp. and *Toxocara* spp. were lower in mature dogs compared to immature dogs. Additional file 6: Figure S4 and Additional file 7: Figure S5 show the forest plots of the estimated prevalences in the sex and age subgroups.

**Table 2:** Estimated pooled prevalences of canine gastrointestinal helminths in sub-Saharan Africa by sex and age

Helminth	Category	Sample size	No. positive	Pooled prevalence	95% CI	I^2^ (%)
Overall prevalence	Male	4279	2569	62	45–78	99
	Female	2965	1547	56	40–71	98
	Immature	1932	1294	74	56–89	98
	Mature	3394	1975	62	42–81	99
	Puppy	400	247	71	45–91	94
	Juvenile	559	286	60	32–85	97
*Ancylostoma* spp.	Immature	1269	519	43	22–64	98
	Mature	1677	587	35	18–55	98
*Toxocara* spp.	Immature	1130	270	25	13–39	95
	Mature	1550	150	9	2–20	97

*Abbreviation*: *CI* confidence interval

There was a high level of heterogeneity in most pooled estimates (I^2^ > 80%) which could not be reduced with subgroup analysis by sub-region, age and sex. Assessment of the funnel plot and doi plot ruled out significant publication bias (Additional file 8: Figure S6).

## Discussion

This study summarised the prevalence of canine gastrointestinal helminths in sub-Saharan Africa. The prevalence of canine helminths in sub-Saharan Africa is high with an estimated pooled prevalence of 71% (95% CI: 63–79%) across 36 studies. The high prevalence of gastrointestinal helminths may be due to inadequate deworming of dogs [17, 19, 23, 26, 35, 38, 40, 41, 43, 53]. The highest prevalence was recorded for *Ancylostoma* spp. These findings are comparable with studies in Portugal [57], Mexico [58, 59], Brazil [60] and Argentina [61]. However, other studies in the Czech Republic [62], Poland [63], Canada [64], Denmark [65] and southern Wisconsin [66] reported *Toxocara* spp. as the most prevalent helminth. These findings suggest that *Ancylostoma* spp. and *Toxocara* spp. are more prevalent in the tropical/sub-tropical and temperate regions, respectively. *Dipylidium caninum* was the most prevalent cestode. This is in agreement with other studies conducted in China [26], Mexico [58, 59], Brazil [60] and Poland [63].

The estimated pooled prevalence of helminths in males was higher than in females. This result is not in agreement with other studies [58, 66–68]. A possible reason for the male-biased helminth prevalence may be the high *Ancylostoma* spp. prevalence. Male-biased *Ancylostoma* prevalences have been reported in some studies [61, 66, 69]. It is possible that male-biased helminth prevalence may occur when the prevalence of *Ancylostoma* spp. is higher than other helminths, as seen in this study. Another possible reason for the male-biased prevalence is the low deworming and neutering prevalences in the dog populations included in this study. Dogs in sub-Saharan Africa receive limited veterinary care and are irregularly dewormed and rarely neutered [26, 70]. Regular deworming and high neutering may significantly reduce the influence of sex on helminth susceptibility in studies that did not demonstrate male-biased prevalence. Unfortunately, the effect on sex on the prevalence of specific helminth genera could not be investigated as the data was not provided in the primary studies.

The overall estimated pooled prevalence of canine intestinal helminths was higher in young dogs than in adults. These results are in agreement with other studies that demonstrated a higher overall prevalence in young animals [67, 68, 71]. Higher prevalence in immature animals has been attributed to lower immune competence in this age group [69]. It would have been interesting to investigate the effect of both age and sex on helminth prevalence; however, this data was not reported in the primary studies.

This study has many limitations. First, the data displayed a large degree of heterogeneity across the studies across and sub-regions. Secondly, the studies were conducted in only eight countries: Nigeria, Ethiopia, Tanzania, Gabon, Ghana, Cameroon, Zambia and South Africa. Moreover, Nigeria and Ethiopia were overrepresented. Therefore, the results may not reflect the real situation in the region. The lack of studies from the majority of countries in sub-Saharan Africa may be due to limited research on canine diseases conducted in the region. Alternatively, it may be due to studies not being published in journals accessible online. Another limitation of this study is the incomplete information provided by some studies. For example, some studies provided limited information on study area while other studies did not report the helminths to species level; consequently, the results could not be further analysed to identify the source of the heterogeneity.

## Conclusions

Despite the limitations of the study, the results indicate that the canine gastrointestinal helminths are highly prevalent in sub-Saharan Africa and there is a need for regular deworming programmes to improve the health status of the dogs and minimise the potential health risk to humans.

## Additional files

Additional file 1: Quality assessment checklist. (DOCX 14 kb)

Additional file 2: **Table S1.** Summary of studies included in the meta-analysis. (DOCX 43 kb)

Additional file 3: **Figure S1.** Frequency distribution of the characteristics of eligible studies. (TIFF 48 kb)

Additional file 4: **Figure S2.** Frequency distribution of canine gastrointestinal helminths. (TIFF 117 kb)

Additional file 5: **Figure S3.** Forest plots of the prevalence estimates. **a** *Ancylostoma* spp. **b** *Toxocara* spp. **c** *Dipylidium caninum.* **d** *Taenia* spp. **e** *Strongyloides* spp. **f** *Uncinaria stenocephala.* **g** *Trichuris vulpis.* **h** *Spirocerca lupi.* **i** *Echinococcus* spp. **j** T*oxascara* spp. **k** Overall prevalence. (TIFF 1424 kb)

Additional file 6: **Figure S4.** Forest plots of the prevalence estimates of gastrointestinal helminths in male (**a**) and female (**b**) dogs. (TIFF 217 kb)

Additional file 7: **Figure S5.** Forest plots of the prevalence estimates of gastrointestinal helminths in: **a** mature dogs; **b** immature dogs; **c** juveniles; **d** puppies. **e** *Ancylostoma* spp. in mature dogs. **f** *Ancylostoma* spp. in immature dogs. **g** *Toxocara* spp. in mature dogs. **h** *Toxocara* spp. in immature dogs. (TIFF 439 kb)

Additional file 8: **Figure S6.** Assessment of publication bias. **a** Funnel plot of the double arcsine transformed prevalence estimates of gastrointestinal helminths in dogs. **b** Doi plot the double arcsine transformed prevalence of gastrointestinal helminths in dogs (LFK index: 0.11). (TIFF 184 kb)

Additional file 9: Prevalence data sets. (XLSX 32 kb)
