# Supplementary material for: Epidemiology of canine gastrointestinal helminths in sub-Saharan Africa
Source: Parasit Vectors. 2018 Feb 20;11:100. doi: 10.1186/s13071-018-2688-9 (PMC5819185; doi:10.1186/s13071-018-2688-9)
Supplement: Supplementary file 1 — Quality assessment checklist. (DOCX 14 kb) [file 13071_2018_2688_MOESM1_ESM.docx]

**Quality assessment checklist**

The following items were examined and given a score based on a simple scale system (1 for ''yes'', 0 for ''no'').

1. Was the research objective clearly stated?
2. Was the sampling area clearly described with reference to the location, climate and level of development (rural, peri-urban or urban)?
3. Was the period of the study stated?
4. Was the target sample a close representation of the general population?
5. Was some form of random selection used to select the samples
6. Was a minimum sample size calculated?
7. Were the sample processing and diagnostic method clearly described?
8. Were the subjects categorised by sex?
9. Were the subjects categorised by ownership and movement restriction and were the categories clearly defined?
10. Were the subjects categorised by age and were the age categories clearly defined?

The quality index score for each study was calculated by dividing the study quality score by 10.
