# Supplementary material for: Epidemiology of canine gastrointestinal helminths in sub-Saharan Africa
Source: Parasit Vectors. 2018 Feb 20;11:100. doi: 10.1186/s13071-018-2688-9 (PMC5819185; doi:10.1186/s13071-018-2688-9)
Supplement: Supplementary file 2 — Table S1. Summary of studies included in the meta-analysis. (DOCX 43 kb) [file 13071_2018_2688_MOESM2_ESM.docx]

Table S1 Summary of studies included in the meta-analysis

| Study | Country | Sample size | Sample type | Processing method | Quality score | Quality index score |
| --- | --- | --- | --- | --- | --- | --- |
| [Abere et al., 2013](file:///C:\Users\NOZYECHI%20CHIDUMAYO\Dropbox\Table%201%20Epidemiology%20of%20canine%20helminthiasis%20in%20Sub-Saharan%20Africa.xlsx#Sheet2!_ENREF_1) [[16](#_ENREF_16)] | Ethiopia | 430 | Faecal | Sedimentation and floatation | 7 | 0.7 |
| [Adedoja et al., 2014](file:///C:\Users\NOZYECHI%20CHIDUMAYO\Dropbox\Table%201%20Epidemiology%20of%20canine%20helminthiasis%20in%20Sub-Saharan%20Africa.xlsx#Sheet2!_ENREF_2) [[17](#_ENREF_17)] | Nigeria | 108 | Faecal | Sedimentation | 6 | 0.6 |
| [Alexander et al., 2016](file:///C:\Users\NOZYECHI%20CHIDUMAYO\Dropbox\Table%201%20Epidemiology%20of%20canine%20helminthiasis%20in%20Sub-Saharan%20Africa.xlsx#Sheet2!_ENREF_3) [[18](#_ENREF_18)] | Madagascar | 33 | Faecal | Floatation | 4 | 0.4 |
| [Amissah-Reynolds et al., 2016](file:///C:\Users\NOZYECHI%20CHIDUMAYO\Dropbox\Table%201%20Epidemiology%20of%20canine%20helminthiasis%20in%20Sub-Saharan%20Africa.xlsx#Sheet2!_ENREF_4) [[19](#_ENREF_19)] | Ghana | 154 | Faecal | Floatation | 7 | 0.7 |
| [Anosike et al., 2004](file:///C:\Users\NOZYECHI%20CHIDUMAYO\Dropbox\Table%201%20Epidemiology%20of%20canine%20helminthiasis%20in%20Sub-Saharan%20Africa.xlsx#Sheet2!_ENREF_6) [[20](#_ENREF_20)] | Nigeria | 554 | Faecal | Not described | 2 | 0.2 |
| [Anosike et al., 2006](file:///C:\Users\NOZYECHI%20CHIDUMAYO\Dropbox\Table%201%20Epidemiology%20of%20canine%20helminthiasis%20in%20Sub-Saharan%20Africa.xlsx#Sheet2!_ENREF_5) [[21](#_ENREF_21)] | Nigeria | 284 | Faecal | Not described | 1 | 0.1 |
| [Awoke et al., 2011](file:///C:\Users\NOZYECHI%20CHIDUMAYO\Dropbox\Table%201%20Epidemiology%20of%20canine%20helminthiasis%20in%20Sub-Saharan%20Africa.xlsx#Sheet2!_ENREF_7) [[22](#_ENREF_22)] | Ethiopia | 326 | Faecal | Floatation | 7 | 0.7 |
| [Bwalya et al., 2011](file:///C:\Users\NOZYECHI%20CHIDUMAYO\Dropbox\Table%201%20Epidemiology%20of%20canine%20helminthiasis%20in%20Sub-Saharan%20Africa.xlsx#Sheet2!_ENREF_8) [[23](#_ENREF_23)] | Zambia | 452 | Faecal | Floatation | 5 | 0.5 |
| [Davoust et al., 2008](file:///C:\Users\NOZYECHI%20CHIDUMAYO\Dropbox\Table%201%20Epidemiology%20of%20canine%20helminthiasis%20in%20Sub-Saharan%20Africa.xlsx#Sheet2!_ENREF_10) [[24](#_ENREF_24)] | Gabon | 198 | Faecal | Floatation | 2 | 0.2 |
| [Degefu et al., 2011](file:///C:\Users\NOZYECHI%20CHIDUMAYO\Dropbox\Table%201%20Epidemiology%20of%20canine%20helminthiasis%20in%20Sub-Saharan%20Africa.xlsx#Sheet2!_ENREF_11) [[25](#_ENREF_25)] | Ethiopia | 334 | Faecal | Floatation | 8 | 0.8 |
| [Selasie et al., 2013](file:///C:\Users\NOZYECHI%20CHIDUMAYO\Dropbox\Table%201%20Epidemiology%20of%20canine%20helminthiasis%20in%20Sub-Saharan%20Africa.xlsx#Sheet2!_ENREF_32) [[26](#_ENREF_26)] | Ethiopia | 448 | Faecal | Sedimentation and floatation | 9 | 0.9 |
| [Edet et al., 2014](file:///C:\Users\NOZYECHI%20CHIDUMAYO\Dropbox\Table%201%20Epidemiology%20of%20canine%20helminthiasis%20in%20Sub-Saharan%20Africa.xlsx#Sheet2!_ENREF_12) [[27](#_ENREF_27)] | Nigeria | 104 | Faecal | Sedimentation and floatation | 5 | 0.5 |
| [Edosomwan & Chinweuba, 2012](file:///C:\Users\NOZYECHI%20CHIDUMAYO\Dropbox\Table%201%20Epidemiology%20of%20canine%20helminthiasis%20in%20Sub-Saharan%20Africa.xlsx#Sheet2!_ENREF_13) [[28](#_ENREF_28)] | Nigeria | 150 | Faecal | Floatation | 4 | 0.4 |
| [Zelalem & Mekonnen, 2012](file:///C:\Users\NOZYECHI%20CHIDUMAYO\Dropbox\Table%201%20Epidemiology%20of%20canine%20helminthiasis%20in%20Sub-Saharan%20Africa.xlsx#Sheet2!_ENREF_40) [[29](#_ENREF_29)] | Ethiopia | 384 | Faecal | Sedimentation and floatation | 8 | 0.8 |
| [Gugsa et al., 2015](file:///C:\Users\NOZYECHI%20CHIDUMAYO\Dropbox\Table%201%20Epidemiology%20of%20canine%20helminthiasis%20in%20Sub-Saharan%20Africa.xlsx#Sheet2!_ENREF_14) [[30](#_ENREF_30)] | Ethiopia | 146 | Faecal | Floatation | 6 | 0.6 |
| [Ibidapo, 2005](file:///C:\Users\NOZYECHI%20CHIDUMAYO\Dropbox\Table%201%20Epidemiology%20of%20canine%20helminthiasis%20in%20Sub-Saharan%20Africa.xlsx#Sheet2!_ENREF_15) [[31](#_ENREF_31)] | Nigeria | 310 | Faecal | Floatation | 5 | 0.5 |
| [Johnson et al., 2015](file:///C:\Users\NOZYECHI%20CHIDUMAYO\Dropbox\Table%201%20Epidemiology%20of%20canine%20helminthiasis%20in%20Sub-Saharan%20Africa.xlsx#Sheet2!_ENREF_16) [[32](#_ENREF_32)] | Ghana | 380 | Faecal | Floatation | 5 | 0.5 |
| [Komtangi et al., 2005](file:///C:\Users\NOZYECHI%20CHIDUMAYO\Dropbox\Table%201%20Epidemiology%20of%20canine%20helminthiasis%20in%20Sub-Saharan%20Africa.xlsx#Sheet2!_ENREF_17) [[33](#_ENREF_33)] | Cameroon | 131 | Faecal | Floatation | 1 | 0.1 |
| [Kutdang et al., 2010](file:///C:\Users\NOZYECHI%20CHIDUMAYO\Dropbox\Table%201%20Epidemiology%20of%20canine%20helminthiasis%20in%20Sub-Saharan%20Africa.xlsx#Sheet2!_ENREF_18) [[34](#_ENREF_34)] | Nigeria | 1000 | Faecal | Sedimentation and floatation | 2 | 0.2 |
| [Magaji et al., 2012](file:///C:\Users\NOZYECHI%20CHIDUMAYO\Dropbox\Table%201%20Epidemiology%20of%20canine%20helminthiasis%20in%20Sub-Saharan%20Africa.xlsx#Sheet2!_ENREF_19) [[35](#_ENREF_35)] | Nigeria | 52 | Faecal | Floatation | 4 | 0.4 |
| [Mahmuda et al., 2012](file:///C:\Users\NOZYECHI%20CHIDUMAYO\Dropbox\Table%201%20Epidemiology%20of%20canine%20helminthiasis%20in%20Sub-Saharan%20Africa.xlsx#Sheet2!_ENREF_20) [[36](#_ENREF_36)] | Nigeria | 40 | Intestinal contents | Sedimentation and floatation | 2 | 0.2 |
| [Matthew et al., 2016](file:///C:\Users\NOZYECHI%20CHIDUMAYO\Dropbox\Table%201%20Epidemiology%20of%20canine%20helminthiasis%20in%20Sub-Saharan%20Africa.xlsx#Sheet2!_ENREF_21) [[37](#_ENREF_37)] | Nigeria | 400 | Faecal | Sedimentation and floatation | 7 | 0.7 |
| [Mekbib et al., 2013](file:///C:\Users\NOZYECHI%20CHIDUMAYO\Dropbox\Table%201%20Epidemiology%20of%20canine%20helminthiasis%20in%20Sub-Saharan%20Africa.xlsx#Sheet2!_ENREF_22) [[38](#_ENREF_38)] | Ethiopia | 860 | Faecal | Sedimentation and simple floatation | 8 | 0.8 |
| [Merga & Sibhat, 2015](file:///C:\Users\NOZYECHI%20CHIDUMAYO\Dropbox\Table%201%20Epidemiology%20of%20canine%20helminthiasis%20in%20Sub-Saharan%20Africa.xlsx#Sheet2!_ENREF_23) [[39](#_ENREF_39)] | Ethiopia | 384 | Faecal | Sedimentation and floatation | 7 | 0.7 |
| [Minnaar & Krecek, 2001](file:///C:\Users\NOZYECHI%20CHIDUMAYO\Dropbox\Table%201%20Epidemiology%20of%20canine%20helminthiasis%20in%20Sub-Saharan%20Africa.xlsx#Sheet2!_ENREF_24) [[40](#_ENREF_40)] | South Africa | 69 | Faecal | Floatation | 3 | 0.3 |
| [Minnaar et al., 2002](file:///C:\Users\NOZYECHI%20CHIDUMAYO\Dropbox\Table%201%20Epidemiology%20of%20canine%20helminthiasis%20in%20Sub-Saharan%20Africa.xlsx#Sheet2!_ENREF_25) [[41](#_ENREF_41)] | South Africa | 63 | Faecal | Floatation | 3 | 0.3 |
| [Mukaratirwa & Singh, 2010](file:///C:\Users\NOZYECHI%20CHIDUMAYO\Dropbox\Table%201%20Epidemiology%20of%20canine%20helminthiasis%20in%20Sub-Saharan%20Africa.xlsx#Sheet2!_ENREF_26) [[42](#_ENREF_42)] | South Africa | 240 | Faecal | Sedimentation | 4 | 0.4 |
| [Nwoha & Ekwuruike, 2011](file:///C:\Users\NOZYECHI%20CHIDUMAYO\Dropbox\Table%201%20Epidemiology%20of%20canine%20helminthiasis%20in%20Sub-Saharan%20Africa.xlsx#Sheet2!_ENREF_27) [[43](#_ENREF_43)] | Nigeria | 210 | Faecal | Sedimentation and floatation | 4 | 0.4 |
| [Odeniran & Ademola, 2013](file:///C:\Users\NOZYECHI%20CHIDUMAYO\Dropbox\Table%201%20Epidemiology%20of%20canine%20helminthiasis%20in%20Sub-Saharan%20Africa.xlsx#Sheet2!_ENREF_28) [[44](#_ENREF_44)] | Nigeria | 104 | Faecal | Floatation and centrifugation | 4 | 0.4 |
| [Okoye et al., 2011](file:///C:\Users\NOZYECHI%20CHIDUMAYO\Dropbox\Table%201%20Epidemiology%20of%20canine%20helminthiasis%20in%20Sub-Saharan%20Africa.xlsx#Sheet2!_ENREF_29) [[45](#_ENREF_45)] | Nigeria | 413 | Faecal | Kato-Katz | 5 | 0.5 |
| [Pam et al., 2013](file:///C:\Users\NOZYECHI%20CHIDUMAYO\Dropbox\Table%201%20Epidemiology%20of%20canine%20helminthiasis%20in%20Sub-Saharan%20Africa.xlsx#Sheet2!_ENREF_30) [[46](#_ENREF_46)] | Nigeria | 100 | Faecal | Sedimentation and Floatation | 3 | 0.3 |
| [Dagmawi et al., 2012](file:///C:\Users\NOZYECHI%20CHIDUMAYO\Dropbox\Table%201%20Epidemiology%20of%20canine%20helminthiasis%20in%20Sub-Saharan%20Africa.xlsx#Sheet2!_ENREF_9) [[47](#_ENREF_47)] | Ethiopia | 455 | Faecal | Sedimentation and floatation | 7 | 0.7 |
| [Salihu et al., 2013](file:///C:\Users\NOZYECHI%20CHIDUMAYO\Dropbox\Table%201%20Epidemiology%20of%20canine%20helminthiasis%20in%20Sub-Saharan%20Africa.xlsx#Sheet2!_ENREF_31) [[48](#_ENREF_48)] | Nigeria | 272 | Faecal | Sedimentation and floatation | 6 | 0.6 |
| [Sowemimo & Asaolu, 2008](file:///C:\Users\NOZYECHI%20CHIDUMAYO\Dropbox\Table%201%20Epidemiology%20of%20canine%20helminthiasis%20in%20Sub-Saharan%20Africa.xlsx#Sheet2!_ENREF_34) [[49](#_ENREF_49)] | Nigeria | 959 | Faecal | Kato-Katz | 5 | 0.5 |
| [Sowemimo, 2009](file:///C:\Users\NOZYECHI%20CHIDUMAYO\Dropbox\Table%201%20Epidemiology%20of%20canine%20helminthiasis%20in%20Sub-Saharan%20Africa.xlsx#Sheet2!_ENREF_33) [[50](#_ENREF_50)] | Nigeria | 269 | Faecal | Kato-Katz | 7 | 0.7 |
| [Swai et al., 2010](file:///C:\Users\NOZYECHI%20CHIDUMAYO\Dropbox\Table%201%20Epidemiology%20of%20canine%20helminthiasis%20in%20Sub-Saharan%20Africa.xlsx#Sheet2!_ENREF_35) [[51](#_ENREF_51)] | Tanzania | 241 | Faecal | Sedimentation and floatation | 4 | 0.4 |
| [Tamerat et al., 2015](file:///C:\Users\NOZYECHI%20CHIDUMAYO\Dropbox\Table%201%20Epidemiology%20of%20canine%20helminthiasis%20in%20Sub-Saharan%20Africa.xlsx#Sheet2!_ENREF_36) [[52](#_ENREF_52)] | Ethiopia | 264 | Faecal | Floatation | 5 | 0.5 |
| [Ugbomoiko et al., 2008](file:///C:\Users\NOZYECHI%20CHIDUMAYO\Dropbox\Table%201%20Epidemiology%20of%20canine%20helminthiasis%20in%20Sub-Saharan%20Africa.xlsx#Sheet2!_ENREF_37) [[53](#_ENREF_53)] | Nigeria | 396 | Faecal | Kato-Katz | 6 | 0.6 |
| [Ugwoke et al., 2011](file:///C:\Users\NOZYECHI%20CHIDUMAYO\Dropbox\Table%201%20Epidemiology%20of%20canine%20helminthiasis%20in%20Sub-Saharan%20Africa.xlsx#Sheet2!_ENREF_38) [[54](#_ENREF_54)] | Nigeria | 70 | Intestinal contents | Washing and decantation | 2 | 0.2 |
| [Yacob et al., 2007](file:///C:\Users\NOZYECHI%20CHIDUMAYO\Dropbox\Table%201%20Epidemiology%20of%20canine%20helminthiasis%20in%20Sub-Saharan%20Africa.xlsx#Sheet2!_ENREF_39) [[55](#_ENREF_55)] | Ethiopia | 120 | Faecal and intestinal contents | Floatation | 5 | 0.5 |
| [Zewdu et al., 2010](file:///C:\Users\NOZYECHI%20CHIDUMAYO\Dropbox\Table%201%20Epidemiology%20of%20canine%20helminthiasis%20in%20Sub-Saharan%20Africa.xlsx#Sheet2!_ENREF_41) [[56](#_ENREF_56)] | Ethiopia | 122 | Faecal and intestinal contents | Sedimentation and floatation | 6 | 0.6 |
