# Supplementary figures and images for: Epidemiology of canine gastrointestinal helminths in sub-Saharan Africa
Source: Parasit Vectors. 2018 Feb 20;11:100. doi: 10.1186/s13071-018-2688-9 (PMC5819185; doi:10.1186/s13071-018-2688-9)

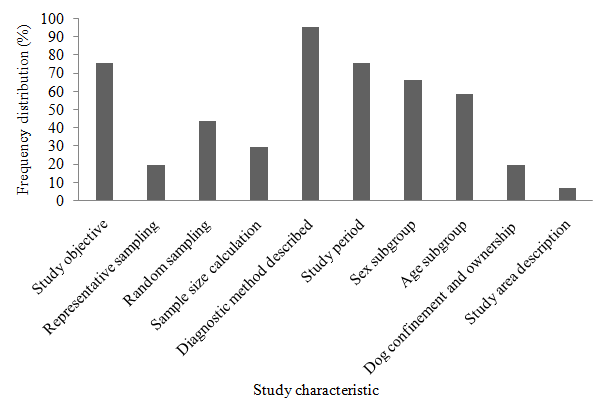

Supplement: Supplementary file 3 — Figure S1. Frequency distribution of the characteristics of eligible studies. (TIFF 48 kb) [file 13071_2018_2688_MOESM3_ESM.tif]

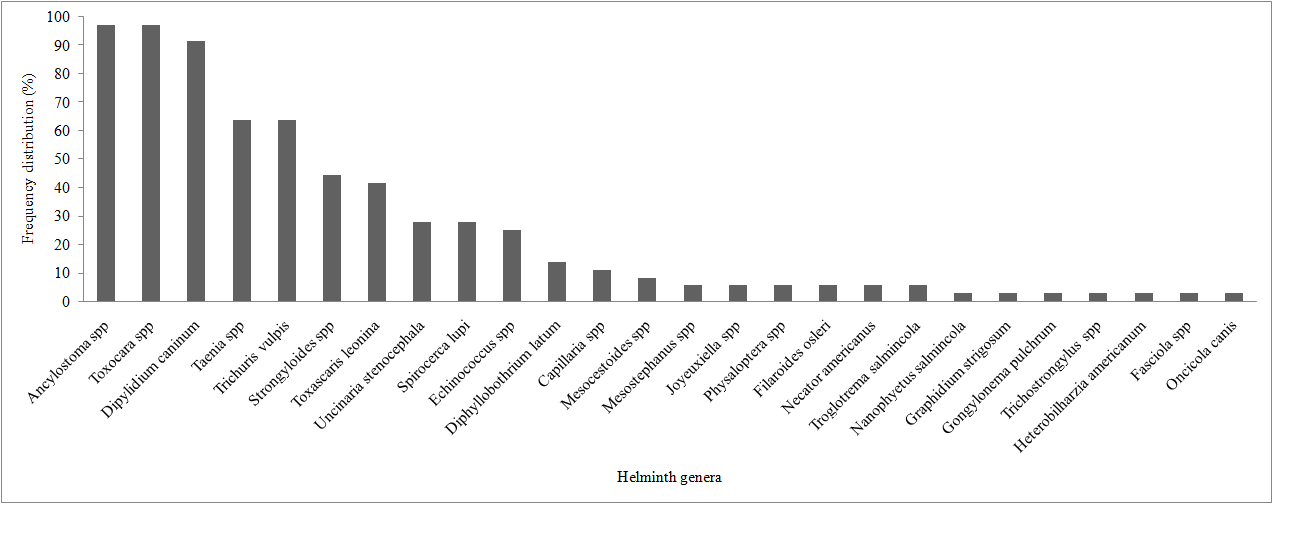

Supplement: Supplementary file 4 — Figure S2. Frequency distribution of canine gastrointestinal helminths. (TIFF 117 kb) [file 13071_2018_2688_MOESM4_ESM.tif]

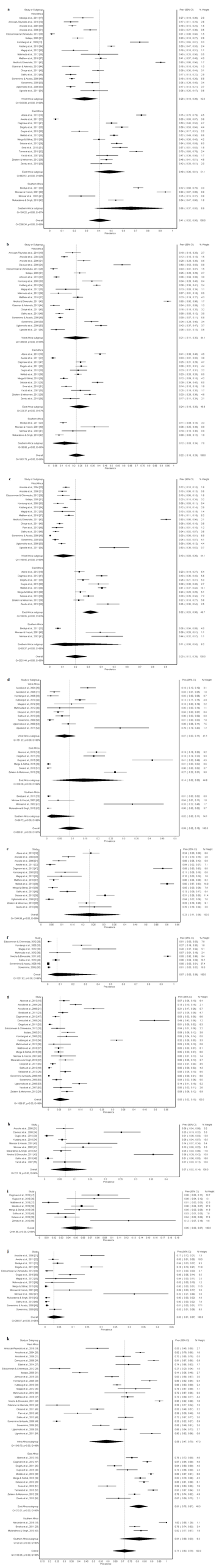

Supplement: Supplementary file 5 — Figure S3. Forest plots of the prevalence estimates. a Ancylostoma spp. b Toxocara spp. c Dipylidium caninum. d Taenia spp. e Strongyloides spp. f Uncinaria stenocephala. g Trichuris vulpis. h Spirocerca lupi. i Echinococcus spp. j Toxascara spp. k Overall prevalence. (TIFF 1424 kb) [file 13071_2018_2688_MOESM5_ESM.tif]

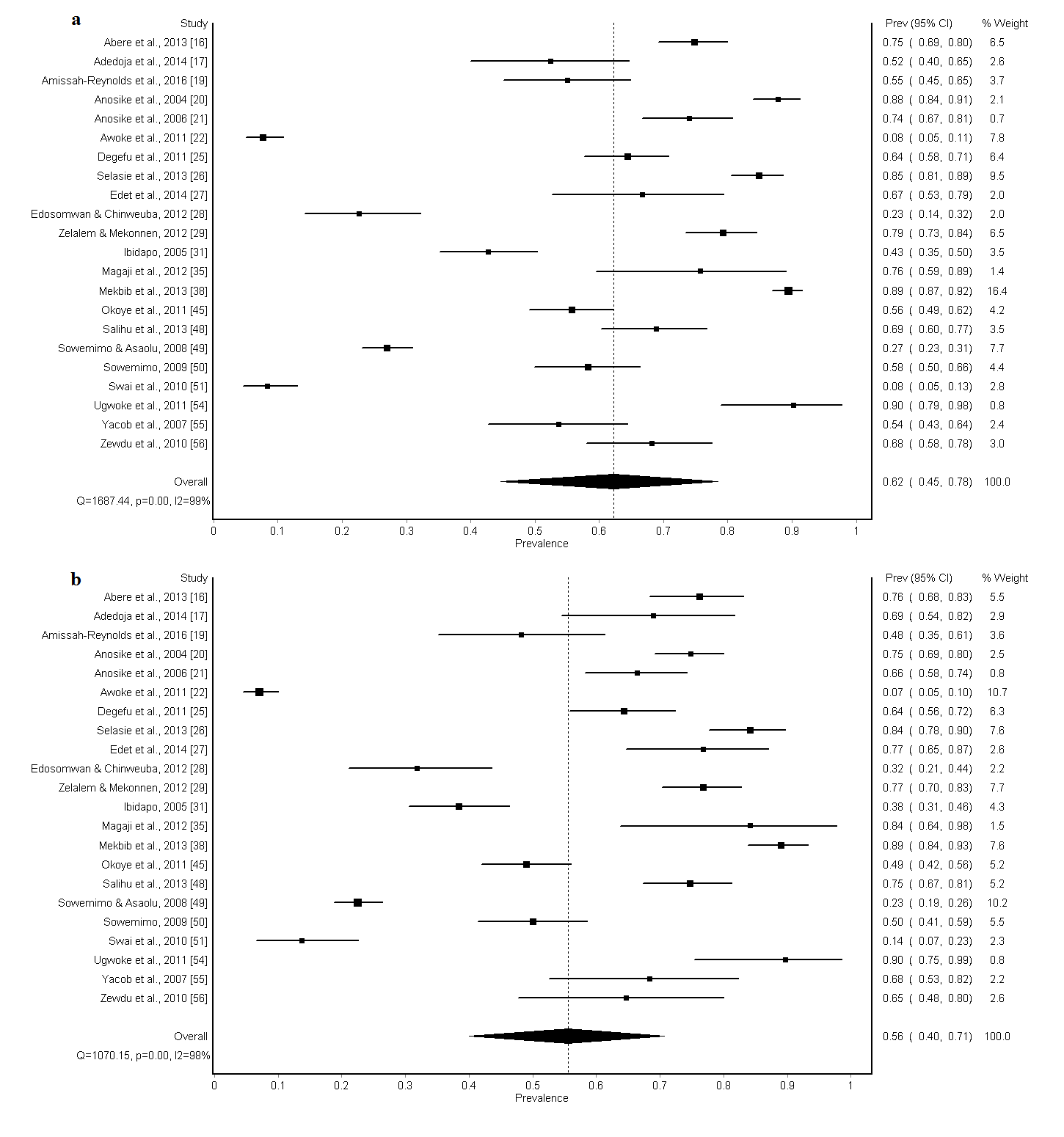

Supplement: Supplementary file 6 — Figure S4. Forest plots of the prevalence estimates of gastrointestinal helminths in male (a) and female (b) dogs. (TIFF 217 kb) [file 13071_2018_2688_MOESM6_ESM.tif]

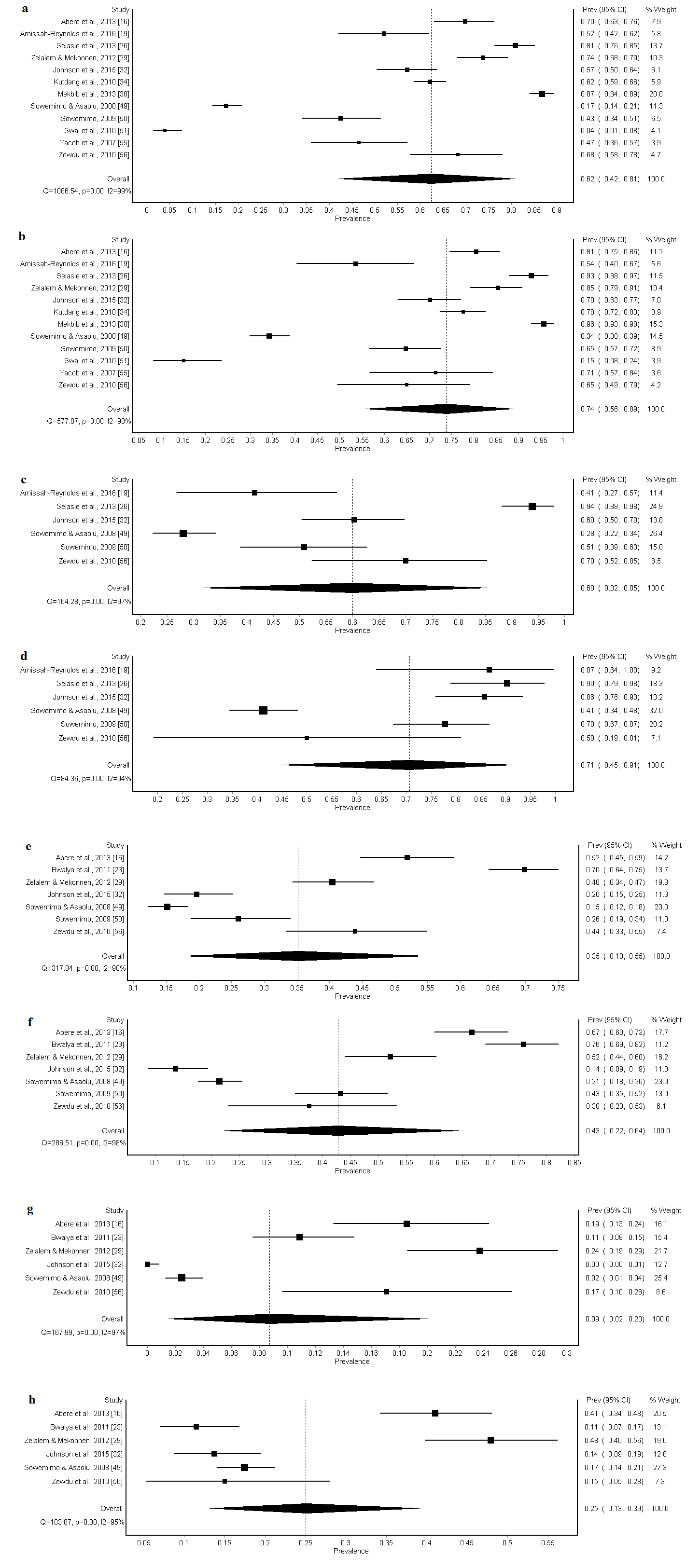

Supplement: Supplementary file 7 — Figure S5. Forest plots of the prevalence estimates of gastrointestinal helminths in: a mature dogs; b immature dogs; c juveniles; d puppies. e Ancylostoma spp. in mature dogs. f Ancylostoma spp. in immature dogs. g Toxocara spp. in mature dogs. h Toxocara spp. in immature dogs. (TIFF 439 kb) [file 13071_2018_2688_MOESM7_ESM.tif]

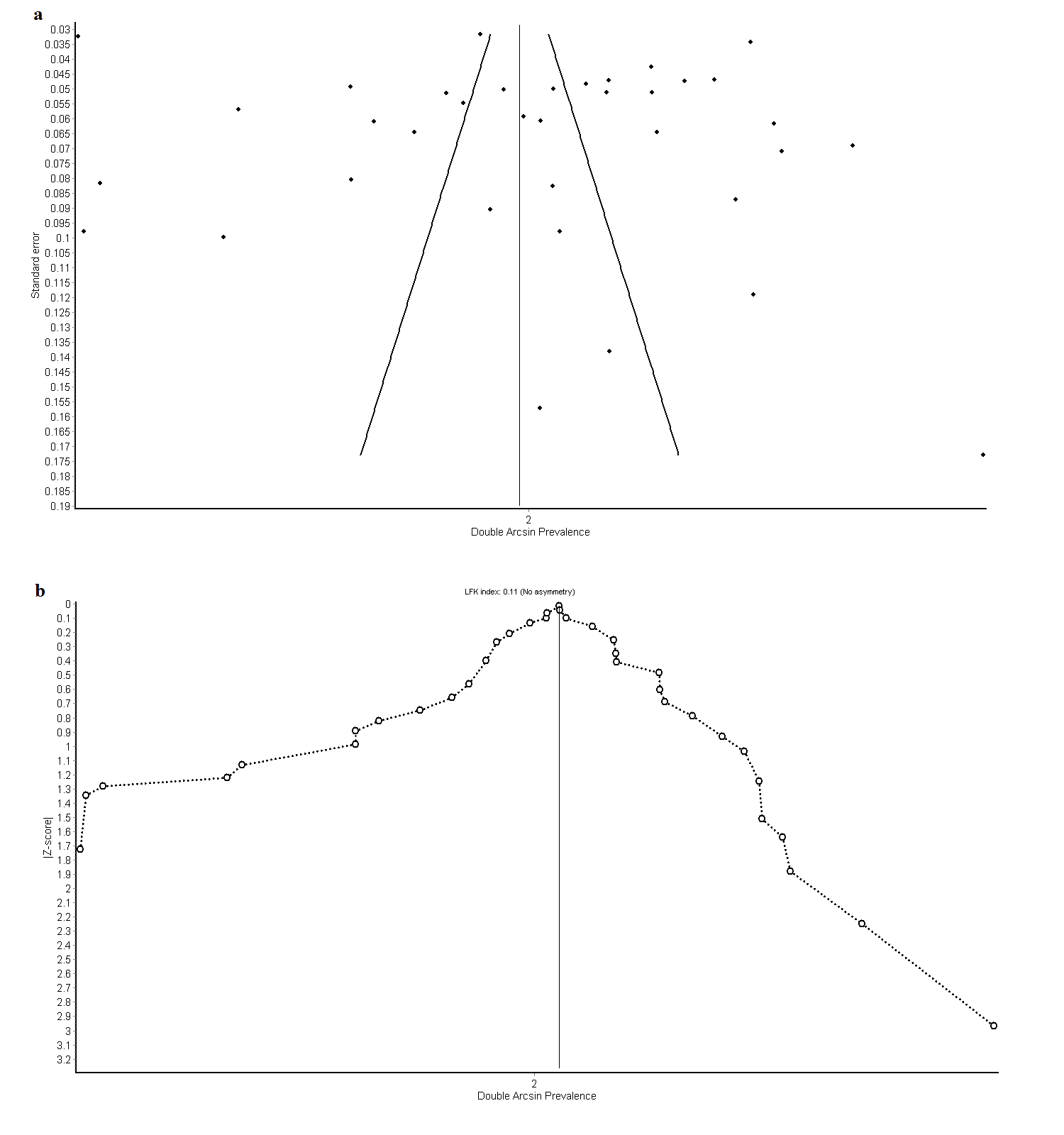

Supplement: Supplementary file 8 — Figure S6. Assessment of publication bias. a Funnel plot of the double arcsine transformed prevalence estimates of gastrointestinal helminths in dogs. b Doi plot the double arcsine transformed prevalence of gastrointestinal helminths in dogs (LFK index: 0.11). (TIFF 184 kb) [file 13071_2018_2688_MOESM8_ESM.tif]
